# Milk Fatty Acid Profile of Holstein Cows When Changed from a Mixed System to a Confinement System or Mixed System with Overnight Grazing

**DOI:** 10.1155/2022/5610079

**Published:** 2022-02-22

**Authors:** Lucía Grille, María L. Adrien, María N. Méndez, Pablo Chilibroste, Laura Olazabal, Juan P. Damián

**Affiliations:** ^1^Departamento de Ciencia y Tecnología de los Alimentos, Facultad de Veterinaria, Universidad de la República, Paysandú, Uruguay; ^2^Departamento de Ciencias Veterinarias y Agrarias, Facultad de Veterinaria, Universidad de la República, Paysandú, Uruguay; ^3^Departamento de Producción Animal y Pasturas, Facultad de Agronomía, Universidad de la República, Paysandú, Uruguay; ^4^Departamento de Desarrollo de Métodos Analíticos, Laboratorio Tecnológico del Uruguay (LATU), Uruguay; ^5^Departamento de Biociencias Veterinarias, Facultad de Veterinaria, Universidad de la República, Montevideo, Uruguay

## Abstract

This study is aimed at comparing the milk fatty acid profile (FAP) of cows that changed from a mixed system (MS) of double grazing plus total mixed ration (TMR) to a total confinement system (TCS, 100% TMR) with cows that changed to another MS with one overnight grazing plus TMR and compare with cows that were kept unchanged in TCS. The diet change was made in the second month of lactation. The milk samples were collected at one (M1-spring) and three months of lactation (M3-summer). Three treatments are as follows (each *n* = 10): confined cows fed with TMR throughout the period (GTMR), cows that changed from MS with double grazing plus TMR in M1 to TCS in M3 (GCHD), and cows that changed from a MS with double grazing plus TMR in M1 to a MS with overnight grazing plus TMR in M3 (GTMR+P). Unlike GTMR+P, GCHD improved milk production after change (increased 14% from M1 to M3), but milk FAP was impaired. In M3, conjugated linoleic acid (C18 : 2-CLA) in GTMR and GCHD was lower than GTMR+P (*p* < 0.05), and linolenic (C18 : 3-n-3) was lower in GCHD than GTMR+P. Maintaining grazing in summer overnight sustained milk fat quality, evidenced by higher C18 : 3 (n-3); C18 : 2 (CLA); and n-6/n-3 ratio than cows that changed to TCS.

## 1. Introduction

Milk is the most complete liquid food of animal origin in nature, and milk and dairy products are among the most important human food [1–3]. Although for decades, ruminant's milk had a negative image due to its high content in saturated fatty acid (SFA) and the relationship of these with cardiovascular diseases [4–7], milk fatty acids are still important nutritional elements. Besides, it has also been reported that milk fat would have beneficial effects on health [8–10]. In this regard, it has been shown that oleic acid (OA; C18 : 1 cis 9), vaccenic fatty acid (VA; C18 : 1 trans 11), and conjugated linoleic acid (CLA) are considered beneficial for human health [11], due to anticarcinogenic, anti-inflammatory, and antiatherogenic effect [12–14]. The main isomer of CLA found in dairy products is rumenic acid (RA; C18 : 2 cis 9 trans 11) [15, 16] which together with the VA are exclusively from ruminants [11]. Furthermore, linolenic fatty acid (FA) (C18 : 3 (n-3)) and linoleic FA (C18 : 2 (n-6)) are the most abundant omega 3 (n-3) and omega 6 (n-6) FA in milk, respectively, both considered essential FA [17]. Moreover, the n-6/n-3 ratio (recommended to be below 4/1) is considered an indicator for nutritional impact of milk fat on human health [18–20]. Therefore, due to the great consumer demand for dairy products, milk composition modification in favor of healthy FA and against SFA and trans FA continues to be a challenge [13].

It is widely reported that animal nutrition is the factor that most influences milk fat composition [21–25]. Fresh pasture has high proportion of unsaturated FA (70–90%) with a large amount of C18 : 2 (n-6) and C18 : 3 (n-3), precursors of stearic acid (C18 : 0), VA, and CLA [26, 27], whereas oilseed contain a high proportion of C18 : 2 [28]. Therefore, the inclusion of fresh forage in mixed dairy systems (pasture plus TMR) improves milk FA profile in favor of those beneficial to human health [25, 27, 29, 30].

The dairy systems in Uruguay are characterized by being pasture-based systems (pasture 55%, supplementation as roughage 19% and concentrates 25%), with an average stocking rate of 1.15 milking cow per hectare, housed in open-sky facilities during the time cows are out of the pasture [31]. In this country, the average milk production in 2020 was 5245 Lt/cow/year [32], and according to Fariña and Chilibroste [31], the “mean productivity was 8831 Lt. per hectare of milking platform (total area of the farm potentially grazable by the milking herd),” data from 2013 to 2017. In such systems, cows are exposed to extreme weather conditions (such as heat stress); notwithstanding, ~63% of dairy farms have access to water near the milking parlor or the paddocks, and although 75% of the farms have natural shade [33], it is usually located on cow trails and not in the paddock or resting areas. In this sense, confinement systems are used to mitigate climatic conditions in summer and minimize its negative effects on milk yield [34, 35], provide independence from forage availability fluctuation [36, 37], and increase total dry matter (DMI) and energy intake per cow to achieve better productive levels [38, 39]. However, beyond the productive advantages provided by confinement systems, the lack of pasture in diet would negatively affect milk fat composition [25, 28, 40, 41], animal welfare [42], and consumer perception of dairy products [20, 43]. Regarding milk fat composition, when cows change from a pasture-based system (grass plus concentrate mix or grass plus total mixed ration: TMR) to 100% TMR, milk SFA increases in detriment of beneficial FA [44, 45]. Meanwhile, when cows switch from TMR to a pasture-based feeding system, the healthy FA increases [41, 44–46]. Hence, during summer, an alternative mixed system consisting of one grazing session at night would improve milk fat composition, while at the same time reducing heat stress negative effects. In addition, although the change from a mixed system to one in confinement in summer improves milk production and biochemical profile in blood, from behavioral point of view, cows fail to adapt in the short term to the lack of pasture, affecting their animal welfare [34]. Although the effect of dietary change on milk FAP when cows switch from pasture to TMR (and vice versa) has been evaluated, to our knowledge, the consequences of changing to a mixed system with one overnight grazing in summer on milk FAP have not been studied. Therefore, we hypothesize that the change from a mixed system with double grazing to overnight grazing during the summer could be an effective management strategy to achieve better milk fat composition than those cows that were changed from mixed to confinement systems or ever-confined cows. The objective of this study was to compare the milk FAP of cows that were changed from a system that combines TMR plus double grazing to a single confinement system (TMR) with cows that changed to a mixed system with only one night grazing and with cows that were kept unchanged in a confinement system (100% TMR).

## 2. Materials and Methods

### 2.1. Location, Animals, and Treatments

The experimental protocol was evaluated and approved by the Comisión Honoraria de Experimentación Animal (CHEA), Universidad de la República, Montevideo, Uruguay (N°149). The study was conducted at a commercial farm located in the Department of Paysandú, Uruguay.

Thirty cows with 2.1 ± 1.2 lactations and an average body weight of 660 kg ± 82.1 kg were used. All cows were under the same management and feeding conditions throughout the 21 days before the expected calving date (prepartum diet). Cows were blocked by calving date, number of lactation, precalving body condition, and live weight and randomly assigned to one of the three following treatments immediately after calving: (1) cows confined and fed with TMR ad libitum (GTMR, *n* = 10) throughout both periods; (2) mixed system cows that changed their diet from double grazing plus 25% TMR of the GTMR (GCHD, *n* = 10) to confinement system (100% TMR); and (3) cows that kept mixed system from double grazing plus 25% TMR of the GTMR to overnight grazing with 35% TMR (GTMR+P, *n* = 10).

Diet change in GCHD and GTMR+P was carried out on November 16th, according to historical records of THI values in the region [34], which corresponds to 70 ± 14 days in milk (DIM). The TMR was offered from 11:00 to 15:00 H in open stalls for all treatments, and cows had free access to water. Feeders covered (70 m × 3 m) with concrete floor and metal roof and an area with dirt floor without roof but with shade in each treatment (50 m × 4 m). All treatments were in the same environment and confinement system but in different and adjacent pens, as described by Grille et al. [34]. The drinking troughs were plastic made (3.76 m × 0.76 m × 0.44 m). Before diet change, GCHD and GTMR+P grazed in two sessions after each milking (08:00–11:00 H and 19:00–06:00 H) and were fed with TMR in one session (11:00–15:00 H, until milking in the afternoon) equivalent to 25% of that received by the GTMR. After diet change, GTMR+P cows grazed in one night session (19:00–06:00 H) and were fed with TMR (from 11:00 to 15:00 H, until milking in the afternoon) with an amount equivalent to 35% of that received by GTMR.

The pasture was composed of *Festuca arundinacea* and *Dactylis perseo.* Daily herbage allowance was 40 kg dry matter/cow. Herbage mass (kg DM/ha) to ground level was estimated weekly using the double sampling technique adapted from Haydock and Shaw [47]. The method was calibrated monthly using a 5-point scale with 3 replicates for each point. Herbage allowance was then determined adjusting daily strip area for grazing. On dry matter (DM) basis, the total mixed ration was composed of whole plant sorghum silage (33%), sorghum dry grain (12.5%), citrus pulp (10%), canola expeller (16.5%), sorghum distillery grain by-product (10%), and soybean husk (16%). In addition, a premix of minerals and vitamins (1.3%) and urea (0.2%) was added.

### 2.2. Data Collection, Measurements, and Estimates

Ration components were analyzed by near-infrared spectroscopy (methods 167.03, 42.05, and 984.13) [48]. Neutral detergent fiber (NDF) and acid detergent fiber (ADF) were measured sequentially [49], without sodium sulphite in the neutral detergent solution using an ANKOM200 Fibre Analyzer (ANKOM Technology Corp., Fairport, NY, USA). NDF was assayed without a heat-stable amylase. Both fiber contents were expressed inclusive of residual ash (Table 1). The TMR was formulated according to the National Research Council [50] for a body weight of 600 kg and 40 Lt/d milk production (4% milk fat).

All cows were milked twice a day at 6:30 and 18:30 H. Milk production was individually recorded with Waikato^®^ meters. Milk samples were collected in two moments, one (M1: week 4, 35 ± 15 DIM) and three months of lactation (M3: week 13, 98 ± 14 DIM) for milk composition and FAP (composite sample and representative of both daily milkings). Total mixed ration and pasture samples were taken in the same moments of milk sampling (M1 and M3) (Table 1). Analyzes for the determination of milk fat were performed using LactoScope FT infrared (FTIR) (Delta Instruments, Drachten the Netherlands).

Milk fat was extracted according to Folch et al. [51], and FA methyl esters were prepared by the transmethylation procedure described by IUPAC 2.301 [52]. Fatty acid methyl esters were quantified using a gas chromatograph (Agilent Technologies 6890, Palo Alto, CA, USA) and a mass spectrometer (Agilent Technologies 5973) in electron ionization mode at 200°C with an electron current of 70 electron volts, acquiring spectra over the mass 35–600 daltons at a rate of 0.7 s/scan with an interscan delay of 0.5 ms. The column chromatography was a SP 2560 (Supelco), highly polar biscyanopropyl capillary column (100 m · 0.25 mm i.d. with 0.2-lm film thickness; Supelco, Bellefonte, PA, USA). Helium was used as the carrier gas with a flow rate of 0.5 mL/min. The injector temperature (split ratio of 1 : 50) was set to 250°C. The initial column temperature (120°C) increased at 10°C/min to 175°C and held for 5 min. Finally, column temperature was increased at a rate of 3°C/min to 240°C and held for 30 min [53]. Samples were run in duplicate, and FAME standard (Supelco 47885-U, Bellefonte; 37 FAME from C4 : 0 to C24 : 0) was analyzed at regular intervals for quality control purposes and to determine recovery and correction factors for individual FA. The intra- and interassay coefficients of variation for each analyte measured were on average 3% and 6%, respectively. Milk fat composition is expressed as grams of each individual FA per 100 g of total FA.

### 2.3. Statistical Analyses

Milk yield, fat (% and kg/d), and FAP were analyzed with an ANOVA for repeated measures using the PROC MIXED of SAS (SAS Institute Inc., Cary, NC, USA (2004). The statistical model for milk yield, fat (content and yield), and FAP included the treatment (GTMR, GCHD, and GTMR+P), month (M1 and M3), and interaction between treatment and month (M1 and M3). The original model also included the parity, but as it was not significant, it was removed from the model. Cows in each treatment were considered as a random effect. In all variables, post-calving days were included as covariable. Post hoc comparisons were performed with the Tukey-Kramer test. Results were considered significant with *p* ≤ 0.05 and tendency when the *p* value was between 0.05 and 0.10. Data are presented as mean ± SEM (standard error of the mean).

## 3. Results

### 3.1. Milk Yield and Composition

There was no difference between treatment and month in milk yield, but a significant interaction between treatment and month was observed (Table 2). In M3, milk yield in GTMR and GCHD was higher than in GTMR+P (*p* = 0.04), but there was no difference between treatments in M1. Milk yield increased from M1 to M3 (*p* = 0.05) in GCHD cows, while GTMR+P cows decreased milk yield between these months (*p* = 0.004).

Milk fat content was affected by treatment, month, and their interaction (Table 2). The cows of GTMR had lower milk fat content than GCHD and GTMR+P (*p* < 0.05), while GCHD and GTMR+P showed no difference between each other. Milk fat content decreased from M1 to M3 (*p* = 0.04). In M1, the GTMR cows had lower milk fat content than GCHD and GTMR+P (*p* = 0.01) and in M3 GTMR+P tended to be higher than GTMR (*p* = 0.08), but there were no differences between GCHD and the other treatments in M3. The cows of GCHD decreased from M1 to M3 (*p* = 0.0005), but the GTMR and GTMR+P showed no difference between these months.

Regarding milk fat yield (kg/d), there were no differences between treatments, but month effect and interaction between treatment and month were observed (Table 2). Fat yield decreased from M1 to M3 (*p* = 0.01). In M1, the GTMR cows had lower fat yield than GCHD and GTMR+P cows (*p* = 0.006), but there was no difference between treatments in M3. Fat yield decreased from M1 to M3 in GTMR+P cows (*p* = 0.0001), but there was no change between these months in GCHD and GTMR cows.

### 3.2. Milk Fatty Acid Profile

There was no treatment effect in SFA, MUFA, PUFA, n-6, n-3, trans, de novo FA, and preformed FA (Table 3).

No interaction between treatment and month was observed in SFA and MUFA (Table 3). An interaction between treatment and month was observed for PUFA (Table 3). There was no difference between treatments in M1 and M3. All treatments decreased from M1 to M3 (*p* < 0.001). The n-6 showed interaction between treatment and month (Table 3). In M1, the percentage of n-6 was higher in GTMR than in GCHD and GTMR+P (*p* < 0.001), but GCHD and GTMR+P had no difference. In M3, there was no difference between treatments.

There was interaction between treatment and month in n-3 (Table 3). No difference between treatments was observed during M1. In M3, the GCHD had lower n-3 than GTMR+P (*p* = 0.02, Table 3), while GTMR showed no difference between GCHD and GTMR+P. The n-3 decreased from M1 to M3 in GCHD (*p* = 0.0001), while in GTMR and GTMR+P did not change between months.

In the n-6/n-3 ratio, the treatment effect was observed (Table 3). The GTMR showed higher n-6/n-3 ratio than GCHD and GTMR+P (*p* < 0.001), and GCHD had higher n-6/n-3 than GTMR+P (*p* < 0.001). An interaction between treatment and month was observed for the n-6/n-3 ratio (Table 3). In M1, the GTMR had higher n-6/n-3 ratio than GCHD cows and GTMR+P (*p* < 0.0001), while GCHD and GTMR+P showed no difference. In M3, there was no difference between GTMR and GCHD, but both were higher than GTMR+P (*p* < 0.0001). The n6/n3 ratio of GTMR decreased from M1 to M3 (*p* < 0.0001, Table 3), while GCHD increased from M1 to M3 (*p* < 0.0001), and GTMR+P did not change between months.

There was interaction between treatment and month in de novo FA (Table 3). In each month, no difference between treatments was observed. The percentage of de novo FA increased from M1 to M3 in the GCHD and GTMR+P (*p* < 0.0001). In fact, in M1, capric (C10 : 0) percentages were higher in GTMR than in GCHD (*p* = 0.05), and lauric (C : 12 : 0) was higher in GTMR than in GCHD and GTMR+P (*p* < 0.05), but there were no differences between treatments in M3 (Table 4). These FA (capric and lauric) increased from M1 to M3 in GCHD (*p* < 0.05), and the lauric FA increased from M1 to M3 in GTMR+P (*p* = 0.01).

There was a trend in the interaction between treatment and month in preformed FA (Table 3). Of them, stearic (C18 : 0), oleic (C18 : 1 cis), linoleic (C18 : 2 cis), linoelaidic (C18 : 2 trans), CLA (C18 : 2), and linolenic (C18 :  3 (n-3)) showed no effect of treatment, but there was an interaction between treatment and month (Table 4). There was no difference in stearic FA (C18 : 0) between treatments.

In M1, GTMR had higher linoleic (C18 : 2 cis (n-6)) than GCHD and GTMR+P (*p* < 0.01), while GCHD and GTMR+P cows showed no difference.

The percentage of C18 : 2 trans was lower in GTMR than in GCHD and GTMR+P (*p* < 0.05). In GCHD, the percentage was lower than in GTMR+P (*p* = 0.02). In M3, there were no differences between treatments in both, C18 : 2 cis and trans (Table 4). The conjugated linoleic acid (C18 : 2) and linolenic (C18 : 3 (n-3)) showed no differences between treatments in M1, while in M3, the percentage of both FA in GCHD was lower than in GTMR+P (*p* < 0.05). Linolenic (C18 : 3 (n-3)) decreased from M1 to M3 in GCHD and GTMR+P cows (*p* < 0.0001). Stearic (C18 : 0), linoleic (C18 : 2 cis), and CLA (C18 : 2) decreased from M1 to M3 in the three treatments (*p* < 0.01). C18 : 1 cis and C18 : 2 trans decreased from M1 to M3 in GCHD and GTMR+P (*p* < 0.01), but in GTMR had no difference between months.

There was month effect in SFA, MUFA, PUFA, n-6, n-3, trans, de novo FA, and preformed FA (Table 3). Saturated FA, as well as de novo and mixed origin FA, increased from M1 to M3 (*p* < 0.001), while MUFA, PUFA, n-6, n-3, and preformed FA decreased from M1 to M3 (*p* = 0.004). The saturated FA increased from M1 to M3, mainly due to C11 : 0, C12 : 0, C13 : 0, C14 : 0, and C15 : 0 FA increase (Table 4). Meanwhile, MUFA decreased from M1 to M3, largely explained by C18 : 1 decrease (*p* < 0.001), and PUFA decreased as a result of C18 : 2 cis (n-6), C18 : 2 trans, C18 : 2 CLA, C18 : 3 (n-3), and n-6 which also decreased from M1 to M3 (*p* ≤ 0.04) (Table 4).

## 4. Discussion

The change from a system that combines double grazing and total mixed ration to a single confinement system (GCHD) or to a mixed system with overnight grazing (GTMR+P) during summer affected milk yield, milk fat content and yield (% and kg/d), and FAP. In the first case (GCHD), milk yield was improved, but milk FAP quality was impaired, in agreement with Pastorini et al. [40], Salado et al. [54], and Mendoza et al. [55]. The higher milk yield in cows fed with TMR could be due in part to the higher total DM and energy intake achieved in a more nutrient-concentrated diet [54, 56, 57]. In relation to milk fat, although the content was diminished with diet change, fat yield (kg/d) did not change possibly by the improvement in milk production. In the second case (GTMR+P), cows showed lower milk yield from M1 to M3, while maintaining a healthier milk FAP for humans after the change (M3). The inability to maintain the productive level in summer (M3) could be due to the worst environmental condition (heat stress) [34], which could also decrease pasture quality [58, 59]. In addition, during summer (M3), pasture NDF and ADF content increased and CP content decreased in comparison with spring (M1). According to these results, it is suggested an advanced phenological stage in plants (decrease in green leaf in relation to stem) which could lead to a decrease in digestibility [60], lower pasture DMI, and lower milk production. Therefore, despite the change in management to reduce heat stress effects in summer (i.e., one overnight grazing and increase of TMR proportion in the diet), cows were not able to maintain their performance.

When comparing the treatments that changed their management strategy in summer (GCHD and GTMR+P), differences in milk FAP were observed, mainly in some FA considered healthy for human consumption. The cows that accessed to overnight grazing during summer (GTMR+P) achieved higher content of healthy FA (e.g., n-3, C18 : 2 (CLA), and C18 : 3) compared to those that changed to a confinement system (GCHD). In addition, GTMR+P showed higher C18 : 2 (CLA) than GTMR in M3. On the other hand, GCHD cows decreased those healthy FA when they were changed to confinement systems. Therefore, cows in a mixed system, even during the summer and overnight grazing, had higher concentration of CLA and linolenic (n-3) in milk than cows in confinement. These results ratify that pasture inclusion in the diet (main source of C18 as linolenic (n-3) and linoleic FA) [46] could increase FA intermediates (VA and RA) and therefore CLA proportion in milk [26, 27]. Our results are also consistent with Morales-Almaraz et al. [61] and Barca et al. [41] who found higher amounts of n-3 in systems that include pasture compared to 100% TMR systems. Besides, in cows with grazing diet (mixed system), lower n-6/n-3 was observed in comparing to confinement system cows. Our results are consistent with studies previously conducted by Barca et al. [41] and Pastorini et al. [40]. In this sense, after change (M3), the GCHD increased the n-6/n-3 ratio to values considered inappropriate for human consumption (above 4/1), while GTMR+P maintained adequate values for human health, as suggested by Simopoulos [18]. Hence, this work highlights the importance of maintaining grazing at night in mixed systems during summer, as it leads to a fatty acid profile considered beneficial for human health, such as high n-3 and C18 : 2 (CLA) content, and low n-6/n-3 ratio [46, 62]. In addition, overnight grazing in a mixed system could be a good management tool as it allows cows to better express their normal behavior, while mitigating heat stress during daylight hours, enabling better welfare [42] and with lower feeding cost in comparison to confinement system [31, 63].

The higher de novo FA percentages in the GCHD and GTMR+P after change (M3) could be caused by lower synthesis inhibition due to lower long-chain FA [16, 64]. Long-chain FAs are potent inhibitors of mammary FA synthesis [24]. These long-chain FAs may reach the mammary gland through circulation from body fat mobilization [64] and diet FA (mainly pasture). In this sense, in both treatments (GCHD and GTMR+P), the preformed FA tended to be higher in M1 than M3, which suggests that there was greater mobilization of reserves in M1 [16, 65, 66]. However, only mixed system cows had greater C18 : 1 (cis) in M1 than in M3, while that GTMR had no difference between months. According to Rukkwamsuk et al. [67], stearic acid (C18 : 0) and oleic acid (C18 : 1cis) are predominant in adipocytes and are released during lipolysis, so we suggest that in our experiment, the cows in mixed system had higher body fat mobilization than confinement cows in M1, which coincides with the inhibition of the synthesis de novo FA. This is consistent with the results reported by Grille et al. [34], where it was observed that mixed system cows during the first month of lactation had greater negative energy balance compared to GTMR cows (evidenced by greater values of NEFA and lower body condition score). Thus, de novo FA synthesis inhibition in mixed systems during M1 could be due to the high proportion of long-chain FA from preformed FAs (lipomobilization) added to those that come directly from the diet (pastures). Moreover, the latter is also noted due to the high content of C18 : 3 in mixed systems than in confinement during M1.

## 5. Conclusion

Maintaining overnight grazing in summer improved milk fat composition, as was evidenced by higher n-3, CLA percentages, especially C18 : 3 (n-3), C18 : 2 (CLA), and n-6/n-3 ratio than those cows that changed from mixed system to confinement system. Therefore, a mixed system with confinement during the day and one overnight grazing could be a good management alternative during the summer, generating a healthier fatty acid profile in milk for consumers.

## Figures and Tables

**Table 1:** Chemical composition and fatty acid profile of total mixed ration (TMR) and pasture in two moments (M1 and M3) of the experiment.

	M1		M3	
Component	TMR	Pasture	TMR	Pasture
DM (%)	66.8	26.7	57.8	21.3
CP (% DM)	16.8	13.6	16.6	10.4
NDF (% DM)	40.4	48.9	35.7	62.0
ADF (% DM)	22.3	24.0	20.5	32.2
Ash (% DM)	6.5	10.1	6.0	10.9
FAs (g/100 g)				
C10 : 0	0.06	nd	nd	nd
C12 : 0	0.09	0.22	nd	nd
C14 : 0	0.41	0.63	0.14	0.55
C15 : 0	0.07	nd	0.05	nd
C16 : 0	14.1	25.2	13.5	29.1
C16 : 1 cis	0.41	0.26	0.63	nd
C16 : 1 trans	0.06	1.98	0.1	1.92
C16 : 2	0.04	nd	nd	nd
C17 : 0	0.15	0.85	nd	0.65
C17 : 1 cis	0.07	nd	0.11	nd
C18 : 0	5.6	13.0	5.6	10.3
C18 : 1 cis	37.3	10.6	41.6	9.3
C18 : 1 trans	0.34	0.09	0.37	nd
C18 : 2 cis (n-6)	36.8	14.7	32.6	17.9
C18 : 2 trans	0.42	0.25	0.43	0.22
C18 : 2 (CLA)	0.05	nd	nd	nd
C18 : 3 (n-3)	2.3	25.0	2.2	23.8
C18 : 3 (n-6)	0.03	0.31	nd	0.34
C20 : 0	0.53	1.12	0.77	1.19
C20 : 1 cis	0.55	0.68	0.54	0.75
C20 : 1 trans	0.05	nd	nd	nd
C20 : 2 cis (n-6)	0.15	nd	0.15	nd
C21 : 0	0.04	0.18	0.05	0.18
C22 : 0	0.29	1.73	0.46	1.55
C23 : 0	0.14	0.22	0.1	0.47
C24 : 0	0.36	1.32	0.45	1.82
C25 : 0	0.07	0.27	0.09	0.11
C26 : 0	0.09	1.51	0.08	nd
C28 : 0	0.03	nd	nd	nd
n-3	2.27	25.0	2.17	23.8
n-6	37.0	15.0	37.1	18.2
Ether extract (g/100)	3.48	2.29	3.14	1.91

M1: one month after calving; M3: three months after calving; CP: crude protein; NDF: neutral detergent fiber; ADF: acid detergent fiber; nd: not detected.

**Table 2:** Effect of treatment (*T*), month (*M*), and interaction between treatment and month (*T*∗*M*) on milk yield and fat (mean ± SEM) in month 1 (M1) and month 3 (M3) of lactation.

		GTMR		GCHD		GTMR+P		SEM	*p* value		
		M1	M3	M1	M3	M1	M3		*T*	*M*	*T*∗*M*
Milk yield (kg/d)		33.89^Aa^	32.34^Aa^	30.88^Aa^	35.30^Ab^	32.48^Aa^	25.99^Bb^	3.5	ns	ns	0.005
Fat	(%)	2.09^Aa^	2.47^Aa^	4.12^Ba^	2.8^ABb^	3.53^Ba^	3.28^Ba^	0.2	0.04	0.04	0.003
	(kg/d)	0.76^Aa^	0.8^Aa^	1.08^Ba^	0.96^Aa^	1.09^Ba^	0.78^Ab^	0.1	ns	0.01	0.013

Treatments: GTMR: cows fed TMR; GCHD: cows diet change; GTMR+P: cows TMR+pasture. When there was significant interaction between treatment and month: differences between treatments in the same month (different capital letters) and difference between months within each treatment (different small letters) indicate *p* < 0.05 were shown. ns: no significance; SEM: standard error of the mean.

**Table 3:** Effect of treatment (*T*), month (*M*), and interaction between treatment and month (*T*∗*M*) on fatty acid profile (FAP) in month 1 (M1) and month 3 (M3) of lactation.

	GTMR		GCHD		GTMR+P		SEM	*p* value		
	M1	M3	M1	M3	M1	M3		*T*	*M*	*T*∗*M*
FA saturation (g/100 g of fat)										
SFA	64.84	71.57	61.01	70.70	63.01	70.40	1.67	ns	<0.001	ns
MUFA	30.01	25.82	35.18	26.83	32.90	26.71	1.54	ns	<0.001	ns
PUFA	5.10^Aa^	2.60^Ab^	3.80^Aa^	2.46^Ab^	4.07^Aa^	2.87^Ab^	0.35	ns	<0.001	<0.001
n-3	0.31^Aa^	0.19^ABa^	0.41^Aa^	0.18^Ab^	0.43^Aa^	0.30^Ba^	0.04	ns	<0.001	0.01
n-6	3.66^Aa^	1.48^Ab^	2.09^Ba^	1.64^Ab^	1.94^Ba^	1.39^Ab^	0.19	ns	<0.001	<0.001
n6/n3	12.30^Aa^	8.52^Ab^	5.02^Ba^	8.95^Ab^	4.47^Ba^	4.72^Ba^	0.37	<0.01	ns	<0.001
Trans	4.93	1.87	4.14	2.12	5.37	3.16	0.50	ns	<0.001	ns
FA origin (g/100 g of fat)										
De novo	24.72^Aa^	25.98^Aa^	18.23^Aa^	25.64^Ab^	19.84^Aa^	25.0^Ab^	1.90	ns	<0.001	0.02
(C4 : 0-C15 : 1)										
Mixed origin	26.73	36.2	29.59	38.95	28.58	36.3	1.20	ns	<0.001	ns
(C16 : 0+C16 : 1)										
Preformed	48.07	37.45	52.31	35.58	51.43	38.49	2.60	ns	<0.001	0.08
(>C17 : 0)										

Treatments: GTMR: cows fed TMR; GCHD: cows diet change; GTMR+P: cows TMR+pasture. When there was significant interaction between treatment and month: differences between treatments in the same month (different capital letters) and difference between months within each treatment (different small letters) indicate *p* < 0.05 were shown. ns: no significance; SFA: saturated; MUFA: monounsaturated; PUFA: polyunsaturated; SEM: standard error of the mean.

**Table 4:** Effect of treatment (*T*), month (*M*), and interaction between treatment and month (*T*∗*M*) on individual milk fatty acid profile in dairy cows in month 1 (M1) and month 3 (M3) of lactation.

	GTMR		GCHD		GTMR+P		SEM	*p* value		
	M1	M3	M1	M3	M1	M3		*T*	*M*	*T*∗*M*
FA (g/100 g of fat)										
C6 : 0	1.67	1.38	1.38	1.26	1.43	1.34	0.30	ns	0.04	ns
C8 : 0	1.48	1.24	1.04	1.07	1.08	1.16	0.20	ns	ns	ns
C10 : 0	3.81^Aa^	3.34^Aa^	2.31^Ba^	2.95^Ab^	2.52^Aa^	3.0^Aa^	0.40	ns	ns	0.03
C11 : 0	0.30^Aa^	0.32^Aa^	0.14^Ba^	0.32^Ab^	0.16^Ba^	0.26^Ab^	0.03	ns	<0.001	<0.001
C12 : 0	3.93^Aa^	3.77^Aa^	2.32^Ba^	3.49^Ab^	2.65^Ba^	3.36^Ab^	0.30	ns	<0.001	0.008
C12 : 1 cis	0.05	0.07	0.03	0.06	0.03	0.07	0.04	ns	<0.001	ns
C13 : 0	0.17^A^	0.20^A^	0.10^B^	0.26^A^	0.12^AB^	0.18^A^	0.02	ns	<0.001	<0.001
C14 : 0	11.16	12.6	9.12	12.7	9.83	12.68	0.80	ns	<0.001	0.09
C14 : 1 cis	0.51	0.84	0.39	0.89	0.43	0.82	0.10	ns	<0.001	ns
C15 : 0	1.63^Aa^	2.22^Ab^	1.38^Aa^	2.60^Ab^	1.57^Aa^	2.16^Ab^	0.10	ns	<0.001	<0.01
C16 : 0	26	34.87	28.04	37.17	27.24	34.8	1.20	ns	<0.001	ns
C16 : 1 cis	0.65	1.26	1.42	1.67	1.2	1.38	0.10	0.03	0.001	ns
C16 : 1 trans	0.08	0.07	0.13	0.11	0.13	0.11	0.01	0.07	<0.001	ns
C17 : 0	1.46	1.66	1.27	1.47	1.42	1.5	0.10	ns	<0.01	ns
C17 : 1 cis	0.11	0.16	0.22	0.23	0.18	0.19	0.02	0.04	ns	ns
C18 : 0	12.5^Aa^	9.43^Ab^	13.86^Aa^	7.43^Ab^	14.6^Aa^	9.62^Ab^	1.00	ns	<0.001	0.02
C18 : 1 cis	24.0^Aa^	21.8^Aa^	29.43^Aa^	22.15^Ab^	26.2^Aa^	21.4^Ab^	1.80	ns	<0.001	0.05
C18 : 1 trans	4.45	1.39	3.47	1.59	4.58	2.55	0.40	ns	<0.001	ns
C18 : 2 cis (n-6)	3.49^Aa^	1.33^Ab^	2.01^Ba^	1.55^Ab^	1.84^Ba^	1.29^Ab^	0.20	0.08	<0.001	<0.001
C18:2 trans	0.34^Aa^	0.34^Aa^	0.49^Ba^	0.34^Ab^	0.59^Ca^	0.42^Ab^	0.04	0.07	<0.001	<0.001
C18 : 2 CLA	0.78^Aa^	0.57^Ab^	0.80^Aa^	0.29^Ab^	1.10^Aa^	0.76^Bb^	0.10	0.05	<0.001	0.02
C18 : 3 (n-3)	0.23^Aa^	0.15^ABb^	0.37^Aa^	0.13^Bb^	0.37^Aa^	0.25^Ab^	0.04	ns	<0.001	<0.001
C20 : 0	0.1	0.09	0.11	0.09	0.12	0.11	0.02	ns	0.04	ns
C20 : 1 cis	0.05	0.04	0.05	0.04	0.05	0.04	0.01	ns	0.04	ns
C20 : 1 trans	0.05	0.07	0.05	0.07	0.06	0.08	0.01	ns	<0.001	ns
C20 : 3 cis	0.07^Aa^	0.05^Ab^	0.04^Ba^	0.05^Ab^	0.05^Ba^	0.05^Aa^	0.01	ns	ns	<0.001
C20 : 4 (n-6)	0.18	0.17	0.08	0.09	0.1	0.11	0.02	ns	ns	ns
C22 : 0	0.11	0.09	0.04	0.03	0.07	0.06	0.02	ns	0.02	ns

Treatments: GTMR: cows fed TMR; GCHD: cows diet change; GTMR+P: cows TMR+pasture. When there was significant interaction between treatment and month: differences between treatments in the same month (different capital letters) and difference between months within each treatment (different small letters) indicate *p* < 0.05 were shown. ns: no significance; SEM: standard error of the mean.

## Data Availability

The data that support the findings of this study are available from the corresponding author upon reasonable request.
